# Molecular Diagnosis of 34 Japanese Families with Leber Congenital Amaurosis Using Targeted Next Generation Sequencing

**DOI:** 10.1038/s41598-018-26524-z

**Published:** 2018-05-29

**Authors:** Katsuhiro Hosono, Sachiko Nishina, Tadashi Yokoi, Satoshi Katagiri, Hirotomo Saitsu, Kentaro Kurata, Daisuke Miyamichi, Akiko Hikoya, Kei Mizobuchi, Tadashi Nakano, Shinsei Minoshima, Maki Fukami, Hiroyuki Kondo, Miho Sato, Takaaki Hayashi, Noriyuki Azuma, Yoshihiro Hotta

**Affiliations:** 1 Department of Ophthalmology, Hamamatsu University School of Medicine, Shizuoka, Japan; 2 Department of Ophthalmology and Laboratory for Visual Science, National Center for Child Health and Development, Tokyo, Japan; 3 Department of Ophthalmology, The Jikei University School of Medicine, Tokyo, Japan; 4 Department of Biochemistry, Hamamatsu University School of Medicine, Shizuoka, Japan; 5 Department of Photomedical Genomics, Institute for Medical Photonics Research, Preeminent Medical Photonics Education & Research Center, Hamamatsu University School of Medicine, Shizuoka, Japan; 6 Department of Molecular Endocrinology, National Research Institute for Child Health and Development, Tokyo, Japan; 7 Department of Ophthalmology, University of Occupational and Environmental Health, Fukuoka, Japan

## Abstract

Leber congenital amaurosis (LCA) is a genetically and clinically heterogeneous disease, and represents the most severe form of inherited retinal dystrophy (IRD). The present study reports the mutation spectra and frequency of known LCA and IRD-associated genes in 34 Japanese families with LCA (including three families that were previously reported). A total of 74 LCA- and IRD-associated genes were analysed via targeted-next generation sequencing (TS), while recently discovered LCA-associated genes, as well as known variants not able to be screened using this approach, were evaluated via additional Sanger sequencing, long-range polymerase chain reaction, and/or copy number variation analyses. The results of these analyses revealed 30 potential pathogenic variants in 12 (nine LCA-associated and three other IRD-associated) genes among 19 of the 34 analysed families. The most frequently mutated genes were *CRB1*, *NMNAT1*, and *RPGRIP1*. The results also showed the mutation spectra and frequencies identified in the analysed Japanese population to be distinctly different from those previously identified for other ethnic backgrounds. Finally, the present study, which is the first to conduct a NGS-based molecular diagnosis of a large Japanese LCA cohort, achieved a detection rate of approximately 56%, indicating that TS is a valuable method for molecular diagnosis of LCA cases in the Japanese population.

## Introduction

Leber congenital amaurosis (LCA; MIM 204000) is the most severe inherited retinal dystrophies (IRDs). Its incidence among the general population is estimated to be only three per 100,000. It is known to affect approximately 20% of children attending schools for the blind, and comprises at least 5% of all IRDs^1,2^.

LCA is a genetically and clinically heterogeneous disease, whose clinical features include blindness or severe visual impairment within the first year of life, nystagmus, very poor or absent ocular pursuit, and a severely reduced or absent electroretinogram (ERG). The fundus appearance in LCA patients varies, either appearing relatively normal, or exhibiting obvious pigmentary changes similar to those seen in patients with retinitis pigmentosa (RP). Other signs and symptoms which may or may not be present in patients with LCA include photophobia, night blindness, hyperopia, macular/peripheral retinal abnormalities, keratoconus, and cataracts^3–5^.

LCA is predominantly inherited as an autosomal recessive (ar) trait; however, rare cases of autosomal dominant (ad) LCA have also been reported^5^. A total of 25 LCA-associated genes have been identified to date (https://sph.uth.edu/retnet/; accessed 14th December 2017). Variants in these genes are estimated to cause approximately 70% of all LCA cases^5–8^.

Gene therapies represent a promising treatment option for patients with LCA^9^; however, their application requires the accurate and effective molecular identification of eligible patients. In addition, LCA has many overlapping clinical features to those observed in other IRDs, which can make it difficult to accurately diagnose a patient. Thus, methods for comprehensive variant screening of patients with LCA are urgently required.

In recent years, the development of DNA sequencing technologies such as next generation sequencing (NGS) has enabled the simultaneous detection of multiple known and novel gene variants. The present study thus performed a comprehensive molecular diagnosis of the largest Japanese LCA cohort investigated to date, by using a targeted-NGS (TS) approach to evaluate the mutation spectra and frequency of known LCA-associated genes in Japanese families with a history of the disease.

## Results

### Patient recruitment

In this study, we newly recruited 33 patients from 31 families who were diagnosed with LCA and fulfilled the three criteria (as described in the Methods section). The results of previously reported 6 patients in 3 families were also included^10,11^, resulting in a total of 39 patients in 34 families analysed. Among 34 families, 30 families include isolate cases with LCA, three families include two patients with LCA, and one family include three patients with LCA^10,11^.

### Identification of potential pathogenic variants in 19 families

The target-capture panel used in the present study comprised 445,968 bp derived from 1182 target regions of the 74 genes^10^. The targeted region of each analysed gene included all exons and flanking intronic sequences (i.e. the intronic sequence ±25 bp from each exon boundary), and the coverage rate was 98.98% (details see Supp. Table 1). The analysed target regions achieved an average 223.9 ± 47-fold coverage across the 33 newly recruited samples, and an average of 91.4% and 86.1% of bases in the target regions exhibited 10-fold and 20-fold coverage, respectively. These results indicate that sufficient coverage was achieved for the identification of variants.

The obtained sequence data were analysed using the filtering criteria described in the methods section. Consequently, in 19 of the 34 analysed families, we identified 30 potential pathogenic variants, of which 16 were novel. All potential pathogenic variants identified in this study were confirmed via Sanger sequencing. These included 16 families that harboured variants in nine LCA-associated genes, and three families that exhibited variants in three other IRD-associated genes. The 30 identified variants consisted of 4 nonsense, 9 frameshift^11^, 3 splice site^10,11^ and 14 missense variants^10^ (Tables 1 and 2). The pathogenicity of the novel missense variants was supported by conducted *in silico* prediction analyses (Supp. Table 2).

**Table 1:** Potential pathogenic variants were identified in 19 of the analysed families.

Family ID	Patient ID	Affected gene	Inherit –ance mode	Identified variant	Zygosity	Origin (as per segregation analysis)	ACMG classifi-cation	Reference
EYE42	EYE42	*NMNAT1*	AR	c.1A > G;p.?	Het	Maternal	P	^13^
				c.709C > T;p.(R237C)	Het	Paternal	P	^12,13^
EYE68	EYE68	*CRB1*	AR	c.668dupT;p.(L223Ffs*4)	Het	Maternal	P	Novel
				c.733dupG;p.(A245Gfs*16)	Het	Maternal	P	Novel
				c.1567dupC;p.(L523Pfs*28)	Het	Paternal	P	Novel
EYE69	EYE69	*CEP290*	AR	c.2390delA;p.(K797Sfs*2)	Het	Maternal	P	^43^
				c.6889A > T;p.(K2297*)	Het	Paternal	P	Novel
EYE70	EYE70	*CRX*	AD	c.124G > A;p.(E42K)	Het	*de novo*	P	^15^
EYE115	EYE115	*CRB1*	AR	c.1334_1740del;p.(C445Yfs*8)	Het	Maternal	P	Novel
				c.1576C > T;p.(R526*)	Het	Paternal	P	^42^
EYE121	EYE121	*CRB1*	AR	c.2T > C;p.?	Het	NA^c^	P	Novel
				c.3068T > G;p.(L1023R)	Het	Maternal	L.P	Novel
EYE125	EYE125	*IMPDH1*	AD	c.590A > C;p.(Q197P)	Het	*de novo*	L.P	Novel
EYE139	EYE139	*GUCY2D*	AR	c.2765A > G;p.(Y922C)	Het	Maternal	L.P	Novel
				c.2983C > T;p.(R995W)	Het	Paternal	P	^14^
EYE149	EYE149	*RPGRIP1*	AR	c.799C > T;p.(R267*)	Het	Paternal	P	^15^
				c.1687C > T;p.(R563*)	Het	Maternal	P	Novel
EYE156	EYE156	*PRPH2*	AR	c.730_736delinsCAGCTCCTCCAGACGGGTGCACCAGAC;p.(N244Qfs*19)	Het	Maternal	P	Novel
				c.748T > G;p.(C250G)	Het	Paternal	L.P	Novel
EYE159	EYE159	*NMNAT1*	AR	c.196C > T;p.(R66W)	Het	Maternal	P	^13^
				c.709C > T;p.(R237C)	Het	Paternal	P	^12,13^
JIKEI-145	JU1039	*LRAT*	AR	c.163C > T;p.(R55W)	Het	Paternal	P	^33^
				Exon 1–3 deletion	Het	Maternal	P	Novel
	JU1040	*LRAT*	AR	c.163C > T;p.(R55W)	Het	Paternal	P	^33^
				Exon 1–3 deletion	Het	Maternal	P	Novel
S132	S132	*NMNAT1*	AR	c.196C > T;p.(R66W)	Het	Paternal	P	^13^
				c.709C > T;p.(R237C)	Het	Maternal	P	^12,13^
EYE50	EYE50	*RPGR*	XL	c.977A > C;p.(K326T)	Hemi	Maternal	L.P	Novel
EYE114	EYE114	*RP2*	XL	c.769–2A > G	Hemi	Maternal	P	Novel
EYE187	EYE187	*BEST1*	AD	c.682G > T;p.(D228Y)	Het	Paternal (mosaic)^b^	L.P	Novel
EYE20^a^	EYE20	*RPGRIP1*	AR	c.3565_3571delCGAAGGC;p.(R1189Gfs*7)	Hom	Biparental	P	^42^
	EYE64	*RPGRIP1*	AR	c.3565_3571delCGAAGGC;p.(R1189Gfs*7)	Hom	Biparental	P	^42^
	EYE65	*RPGRIP1*	AR	c.3565_3571delCGAAGGC;p.(R1189Gfs*7)	Hom	Biparental	P	^42^
EYE55^a^	EYE55	*RPGRIP1*	AR	c.1467+1G > T	Het	Paternal	P	^11^
				Exon 17 deletion (c.2710 + 374_2895 + 74del)	Het	Maternal	P	^11,25^
LCA1H^a^	Twin 1	*GUCY2D*	AR	c.2113 + 2_2113 + 3insT	Het	Paternal	P	^10^
				c.2714 T > C;p.(L905P)	Het	Maternal	P	^10^
	Twin 2	*GUCY2D*	AR	c.2113 + 2_2113 + 3insT	Het	Paternal	P	^10^
				c.2714T > C;p.(L905P)	Het	Maternal	P	^10^

^a^Mutation analyses for families EYE20, EYE55, and LCA1H were reported previously^10,11^.

^b^See Supp. Figure 2 for sequencing electropherogram data.

^c^DNA was unavailable for the patient’s father, as he was deceased.

NA, not available; Hom, homozygous; Het, heterozygous; Hemi, hemizygous; P, pathogenic; L.P, likely pathogenic; AD, autosomal dominant; AR, autosomal recessive; XL, X-linked.

**Table 2:** Identified potential pathogenic variants in Leber congenital amaurosis (LCA)- and other inherited retinal dystrophies (IRD)-associated genes.

Disorder	Gene	Accession number	Variant type	Nucleotide change	Location in gene	Patients found to harbour variant	SNP ID	Database listings^a^			
								HGVD	ToMMo	1000 Genomes	ExAC
LCA	*CRB1*	NM_201253.2	Insertion	c.668dupT;p.(L223Ffs*4)	Exon 3	EYE68		0	0	0	0
				c.733dupG;p.(A245Gfs*16)	Exon 3	EYE68		0	0	0	0
				c.1567dupC;p.(L523Pfs*28)	Exon 6	EYE68		0	0	0	0
			Nonsense	c.1576C > T;p.(R526*)	Exon 6	EYE115	rs114342808	0	0.0002	0	4.94 × 10^−5^
			Deletion	c.1334_1740del;p.(C445Yfs*8)	Exon 6	EYE115		0	0	0	0
			Missense	c.2T > C;p.?	Exon 1	EYE121		0	0	0	0
				c.3068T > G;p.(L1023R)	Exon 9	EYE121		0	0.0002	0	0
	*NMNAT1*	NM_022787.3	Missense	c.1A > G;p.?	Exon 2	EYE42		0	0	0	8.26 × 10^−6^
				c.196C > T;p.(R66W)	Exon 3	EYE159, S132		0	0.0002	0	0.0001
				c.709C > T;p.(R237C)	Exon 5	EYE42, EYE159, S132	rs375110174	0.0009	0.0002	0	7.42 × 10^−5^
	*RPGRIP1*	NM_020366.3	Deletion	c.3565_3571delCGAAGGC;p.(R1189Gfs*7)	Exon 22	EYE20, EYE64, EYE65, EYE170		0	0	0	1.66 × 10^−5^
			Splicing	c.1467 + 1G > T	Intron 11	EYE55		0	0	0	0
			Deletion	Exon 17 deletion (c.2710 + 374_2895 + 74del)	Exon 17	EYE55, EYE16, JU0954, JU0955		0	0	0	0
			Nonsense	c.799C > T;p.(R267*)	Exon 5	EYE149	rs554396590	0	0	0.0002	8.36 × 10^−6^
				c.1687C > T;p.(R563*)	Exon 13	EYE149		0	0	0	0
	*GUCY2D*	NM_000180.3	Splicing	c.2113 + 2_2113 + 3insT	Intron 10	LCA1H (Twin 1 and 2)		0	0	0	0
			Missense	c.2714T > C;p.(L905P)	Exon 14	LCA1H (Twin 1 and 2)		0	0	0	0
				c.2765A > G;p.(Y922C)	Exon 14	EYE139		0	0.0003	0	0
				c.2983C > T;p.(R995W)	Exon 16	EYE139	rs61750187	0	0	0	0
	*CEP290*	NM_025114.3	Deletion	c.2390delA;p.(K797Sfs*2)	Exon 23	EYE69	rs781670422	0	0	0	5.10 × 10^−5^
			Nonsense	c.6889A > T;p.(K2297*)	Exon 50	EYE69		0	0	0	0
	*CRX*	NM_000554.4	Missense	c.124G > A;p.(E42K)	Exon 3	EYE70	rs863224863	0	0	0	0
	*IMPDH1*	NM_000883.3	Missense	c.590A > C;p.(Q197P)	Exon 8	EYE125		0	0	0	0
	*LRAT*	NM_004744.4	Missense	c.163C > T;p.(R55W)	Exon 2	JU1039, JU1040	rs527236079	0.0018	0.0025	0	0
			Deletion	Exon 1–3 deletion	Exon 1–3	JU1039, JU1040		0	0	0	0
	*PRPH2*	NM_000322.4	Deletion/ Insertion	c.730_736delinsCAGCTCCTCCAGACGGGTGCACCAGAC;p.(N244Qfs*19)	Exon 2	EYE156		0	0	0	0
			Missense	c.748T > G;p.(C250G)	Exon 2	EYE156		0	0	0	0
Other IRD	*RP2* ^b^	NM_006915.2	Splicing	c.769-2A > G	Intron 2	EYE114		0	0	0	0
	*RPGR* ^b^	NM_000328.2	Missense	c.977A > C;p.(K326T)	Exon 9	EYE50		0	0	0	0
	*BEST1* ^c^	NM_004183.3	Missense	c.682G > T;p.(D228Y)	Exon 6	EYE187		0	0	0	0

^a^Databases include: 1000 Genomes database (1000 Genomes; http://www.1000genomes.org/), Exome Aggregation Consortium database (ExAC; http://exac.broadinstitute.org/), Human Genetic Variation Database (HGVD; http://www.genome.med.kyoto-u.ac.jp/SnpDB/), and Tohoku Medical Megabank Organization database (ToMMo; https://ijgvd.megabank.tohoku.ac.jp/).

^b^*RPGR* and *RP2* mutations have been previously shown to cause X-linked retinitis pigmentosa (RP).

^c^Mutations in *BEST1* gene have been shown to cause Best vitelliform macular dystrophy, autosomal recessive bestrophinopathy, adult-onset vitelliform macular dystrophy, autosomal dominant vitreoretinochoroidopathy, and autosomal dominant RP.

AD, autosomal dominant; AR, autosomal recessive; XL, X-linked.

### Variants in LCA-associated genes

**Figure 1:**
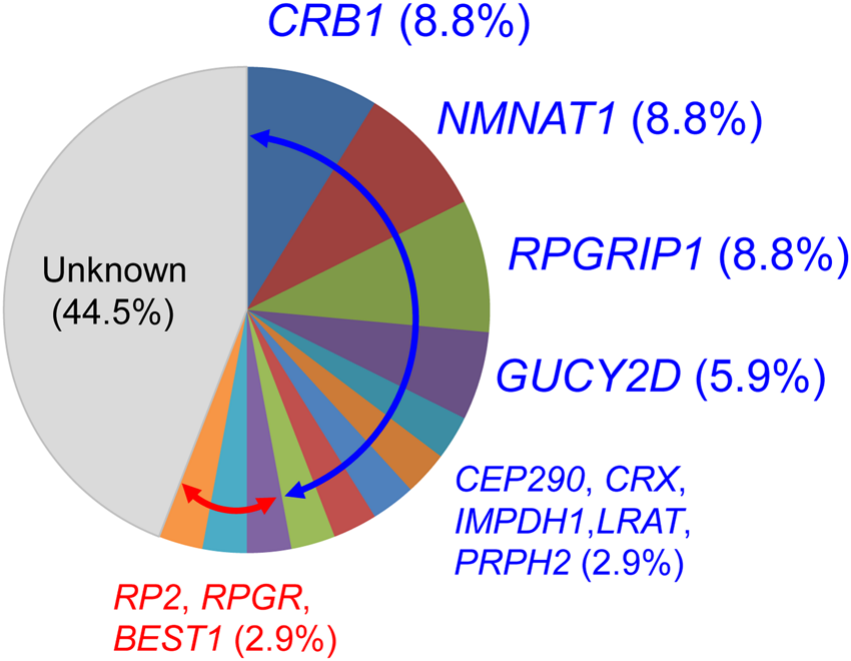
Frequency of identified variants in Leber congenital amaurosis (LCA)-associated genes in the 34 analysed Japanese families. In total, 19 of the 34 analysed families were shown to carry potential pathogenic variants, of which 16 were found to harbour variants in nine LCA- (Blue) and three other IRD-associated (Red) genes, respectively. The most frequently mutated genes in the analysed cohort were *CRB1*, *NMNAT1*, and *RPGRIP1*.

**Figure 2:**
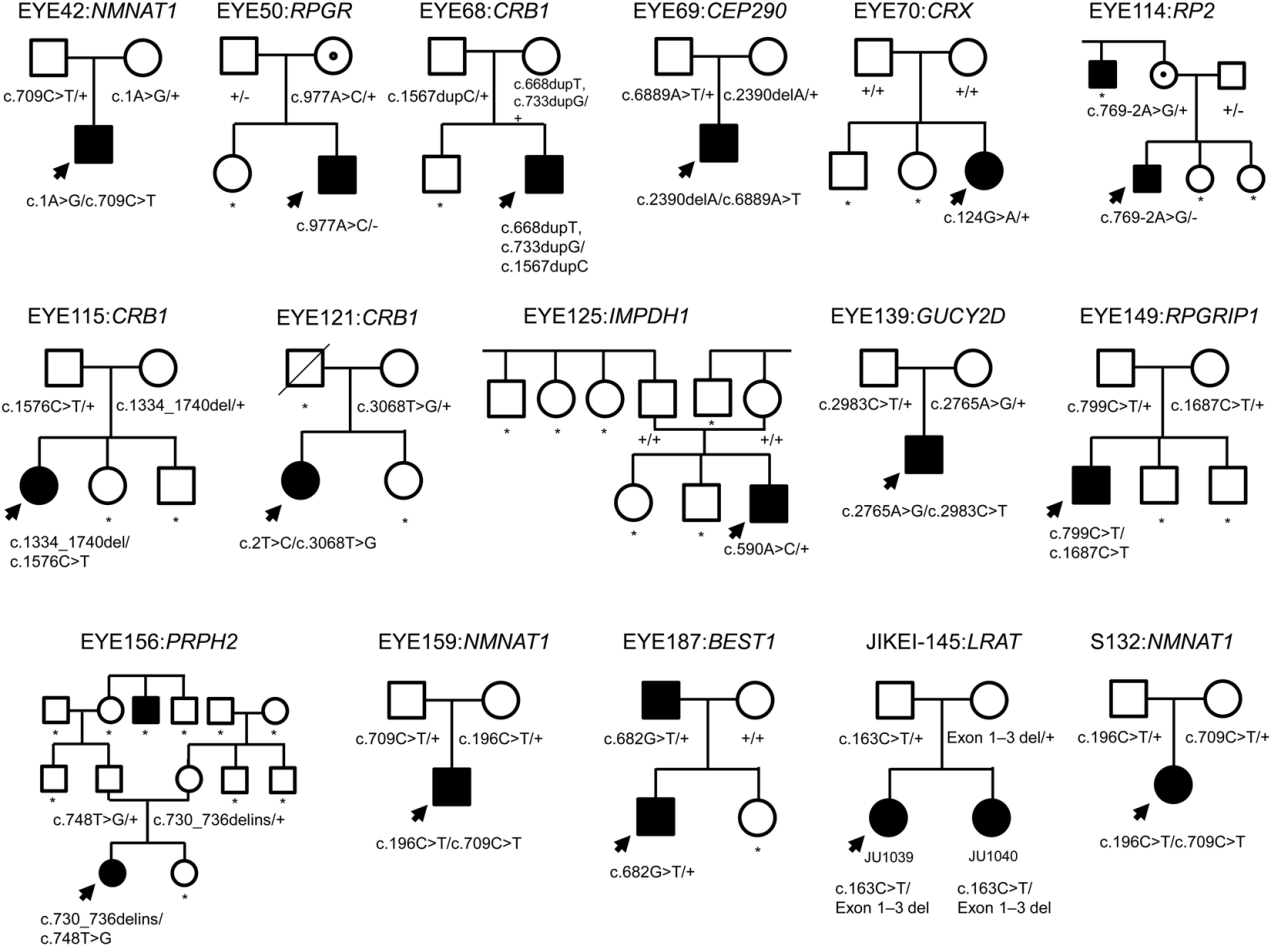
Pedigrees and segregation analyses for the analysed families. The results of the segregation analyses for families EYE20, EYE55, and LCA1H have been previously reported^10,11^. Patient EYE68 harboured three variants, of which both p.(L223Ffs*4) and p.(A245Gfs*16) were maternally inherited, and predicted to produce transcripts likely to be targeted for nonsense-mediated mRNA decay, and thus no final protein product. Both variants were thus considered to be likely pathogenic; however, this was not able to be confirmed by the present study. The parents in family EYE156 were normally sighted at the time of their examinations; however, it is possible that this may be a delayed effect of a late-onset form of retinitis pigmentosa (RP) likely to be induced as a result of their identified heterozygous variants in *PRPH2*, a known autosomal dominant RP-associated gene. The identification code and gene(s) relevant to each family are shown above the pedigree, and the genotype for each family member is shown below their symbol. Probands are indicated by arrows. Square, male; circle, female; black colour, disease-affected; circle with a central black dot, X-linked carrier; slashed symbol, deceased individuals; asterisk, no available DNA sample.

Of the identified potential pathogenic variants in LCA-associated genes, 25 (in 14 families) were identified to occur in seven known ar LCA-associated genes, while two (in two families) were identified to occur in two known ad LCA-associated genes (Tables 1 and 2). The most frequently mutated genes identified by the present study were *CRB1*, *NMNAT1*, and *RPGRIP1*, which were each found to occur in three of the 19 families (Fig. 1). The three identified *NMNAT1* variants (carried by patients EYE42, EYE159, and S132) were missense variants that were previously shown to be pathogenic^12,13^, while the five identified *RPGRIP1* variants, and five of the seven identified *CRB1* variants included both previously reported and novel variants that were all shown to induce a loss-of-function (LOF). Of the remaining seven families, five carried *CEP290*, *LRAT*, *PRPH2*, *CRX*, and *IMPDH1* variants, and two were shown to harbour four *GUCY2D* variants, three of which were missense variants^10,14^. The conducted segregation analyses of 19 of the identified families confirmed that the pathogenic alleles were on different chromosomes^10,11^ (Table 1 and Fig. 2). Although TS data suggested that elder (JU1039) and younger (JU1040) affected sisters in family JIKEI-145 had an apparently homozygous missense variant c.163C > T [p.(R55W)] in exon 3 of the *LRAT*, the conducted segregation analysis revealed that the sisters’ unaffected father had the heterozygous variant and their unaffected mother did not. This finding suggested the possibility that both the sisters had a heterozygous deletion including exon 3 of *LRAT*. In fact, quantitative real-time polymerase chain reaction (qPCR) analysis revealed that both the sisters and their mother harboured a heterozygous deletion that included *LRAT* exons 1–3 (Supp. Figure 1), indicating that the pathogenic two alleles were on different chromosomes (Table 1 and Fig. 2). The patients with LCA in families EYE70 and EYE125 carried a known heterozygous missense variant in *CRX*^15^ and a novel heterozygous missense variant in *IMPDH1*, respectively, both of which occurred *de novo* (Table 1 and Fig. 2), consistent with an ad mode of inheritance.

### Variants in other IRD-associated genes

Mutations in *RPGR* and *RP2* have been previously shown to cause X-linked RP. We found a novel hemizygous missense variant c.977A > C [p.(K326T)] in *RPGR*, and a novel hemizygous splice site variant c.769-2A > G in *RP2* in families EYE50 and EYE114, respectively, which were maternally transmitted (Table 1 and Fig. 2). *BEST1* mutations have been previously shown to cause five clinically distinct retinopathies, including Best vitelliform macular dystrophy, ar bestrophinopathy, adult-onset vitelliform macular dystrophy, ad vitreoretinochoroidopathy, and ad RP^16^. The present study identified a novel heterozygous *BEST1* missense variant c.682G > T [p.(D228Y)] that was carried by the patient with LCA in family EYE187. The patient’s mother was normally sighted, whereas his father had previously been diagnosed with RP. The produced sequencing electropherogram suggested that his father may harbour the variant in a mosaic state (Supp. Figure 2). To evaluate the paternal mosaicism state, it is necessary to perform the segregation analysis using the parental DNA extracted from several tissues. However, the parents did not agree to receive the more elaborate examinations. The p.(D228Y) was a novel missense change at an amino acid residue, where a different missense changes [p.(D228N) and p.(D228H)] were reported to be pathogenic^17–19^. Xu Y *et al*. previously described a patient with LCA (patient ID:LH38 in their manuscript) that exhibited a heterozygous *BEST1* missense variant p.(D228H)^17^. Therefore, it is likely that the p.(D228Y) may cause LCA in the patient.

### Additional mutation screening

Unsolved families, (i.e. those for which potentially pathogenic variant(s) were not detected using the TS approach) were analysed via additional mutation screening (as described in the Methods section). None of the patients in these families carried the c.2991 + 1655A > G intronic variant in *CEP290* that is frequently identified in European, Australian, and Brazilian families with LCA^5,7,8^. Neither were any of the analysed patients shown to harbour rare variants in five recently discovered LCA-associated genes (*CCT2*, *CLUAP1*, *DTHD1*, *GDF6*, and *IFT140*)^20–24^, nor in exon 15 of *RPGR*, which is an alternative exon called ORF15 that contains highly repetitive purine-rich sequences (Supp. Tables 3 and 4).

### TS data reanalysis and Copy number variation (CNV) analyses

The utilised TS approach identified seven patients (EYE16, EYE47, EYE63, EYE152, EYE170, EYE178, and LCA2H) that carried a single heterozygous rare variant in an ar LCA-associated gene (Supp. Table 3). In addition, the heterozygous known *RPGRIP1* exon-17 deletion (c.2710 + 374_2895 + 74del)^11,25^ was identified in families EYE16 and JIKEI-122 via breakpoint-specific PCR (See the Methods section and Supp. Table 4). These eight families harbour a heterozygous variant in LCA-associated gene; this rate is much higher than the occasional carrier rate of ar variants, suggesting that a second variant is likely located within these genes. Therefore, the generated TS data was used to re-search for splice-site variants within 25 bp of the exon-intron boundaries, and for variants in noncoding exonic regions (see Methods section). The results of these analyses did not reveal any second variants within the analysed genes for these families. Meanwhile, eight of these identified patients, (since patient EYE47 was excluded from further analysis due to a lack of sample DNA), were assessed via a multiplex ligation-dependent probe amplification (MLPA) assay. The results of this assay showed no CNV on the alternate allele in any of the analysed patients (Supp. Table 4).

### Patient clinical findings

All enrolled patients met the three criteria (as described in the Methods section), and the 19 patients shown to carry potential pathogenic variants exhibited classical clinical features of LCA. Ophthalmoscopic examinations of the patients revealed various degrees of retinal degeneration, with or without vascular attenuation, macular degeneration, and optic disc pallor. The decimal best-corrected visual acuity (BCVA) in measurable cases ranged from light perception to 0.5.

**Figure 3:**
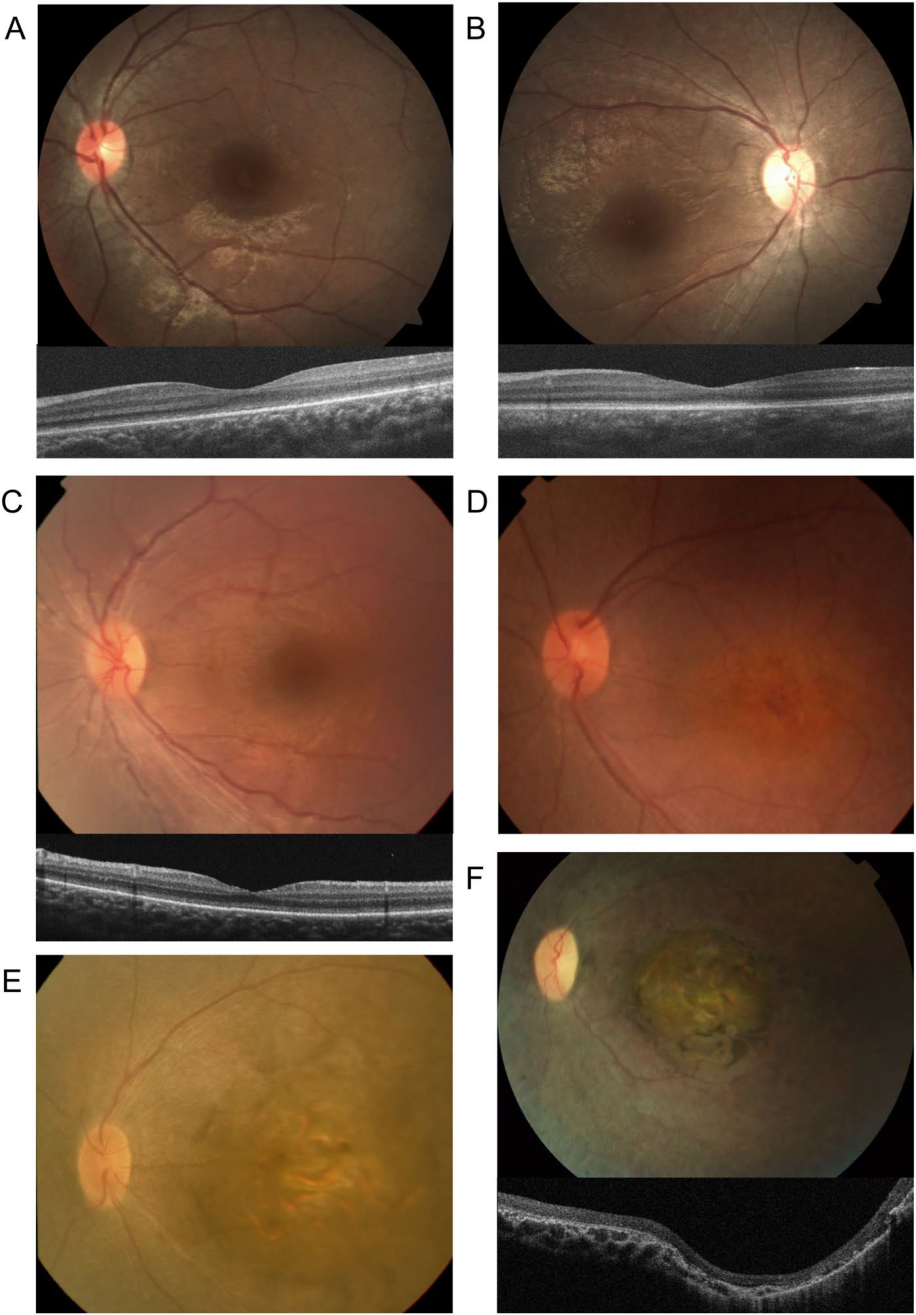
Clinical findings for six patients with Leber congenital amaurosis-associated mutations. The presented fundus photographs revealed that patients **(A)** JU1039 (at the age of 9 years), **(B)** JU1040 (at the age of 6 years), **(C)** EYE139 (at the age of 22 months), **(D)** EYE121 (at the age of 8 years), **(E)** EYE42 (at the age of 18 months), and **(F)** S132 (at the age of 14 years) all exhibited retinal degeneration, diffuse retinal pigment epithelium (RPE) atrophy, and retinal vessel narrowing; however, the degeneration was milder in patients JU1039, JU1040, and EYE139. Additionally, coloboma-like macular atrophy was observed in patients S132 and EYE42. The accompanying optical coherence tomography (OCT) images presented underneath **(A–C)**, and **(F)** revealed an unremarkable ellipsoid zone, but a relatively preserved retinal structure and thickness in patients JU1039, JU1040, and EYE139, as well as both severe retinal thinning and a disrupted retinal structure in patient S132.

Among the families assessed by the present study (Fig. 2), the phenotype data collected for family JIKEI-145 (including patients JU1039 and JU1040) were found to be relatively mild (Fig. 3A,B). The proband, JU1039, was a 9-year-old female that exhibited nyctalopia from early childhood. Her BCVA scores at the time of examination were 0.5 and 0.2 (with hyperopia) in the right and left eye, respectively. Her younger sister, JU1040, was a 6-year-old female that also exhibited nyctalopia from early childhood. Her BCVA score at the time of examination were 0.2 and 0.3 (with hyperopia) in the right and left eye, respectively. Fundus examinations revealed almost normal fundi, with only mild diffuse retinal pigment epithelium (RPE) atrophy, and slight retinal vessel narrowing in both patients; however, full-field ERGs were severely decreased and non-recordable for JU1039 and JU1040, respectively. Goldmann perimetry testing showed preserved peripheral visual fields with V-4e isopters, but decreased central sensitivity in both patients. Together these data represent the first report describing the clinical features of Japanese patients with *LRAT-*associated LCA. Patient EYE139 was found to harbour a *GUCY2D* mutation, and to exhibit almost normal fundi, with only mild retinal degeneration (Fig. 3C). He was a 9-year-old male with autism, and exhibited nystagmus from birth. His visual acuity (measured using a grating acuity card under binocular conditions), was 0.02 (with hyperopia).

Conversely, patient EYE121 was found to carry a *CRB1* mutation, and to display a more severe phenotype (Fig. 3D). She was a 14-year-old female that exhibited nystagmus from birth. Her BCVA scores at the time of examination were 0.3 and 0.06 (with hyperopia) in the right and left eye, respectively. Fundus examinations revealed marked retinal degeneration with maculopathy, slight retinal vessels narrowing, and pigmentation in the midperiphery. Patients EYE42 and S132 were both shown to carry *NMNAT1* mutations, and exhibit very severe phenotypes (Fig. 3E,F). EYE42 was a 16-year-old male with no light perception, while S132 was a 14-year-old female that exhibited only light-perception vision. Both patients exhibited nystagmus from birth, as well as bilateral coloboma-like macular atrophy, diffuse RPE atrophy, and retinal vessel narrowing (identified via the conducted fundus examinations).

## Discussion

In this study, we described the mutation screening in a total of 34 Japanese families with LCA (including three previously described families^10,11^), which we believe is the largest Japanese LCA cohort studied until date. The utilised TS and additional screening methods revealed 30 potential pathogenic variants in 19 of the analysed families. A number of potential pathogenic variants have already been identified in families of various ethnic populations, and several have described variants in Japanese families with LCA^25–30^. However, this study provides the mutation spectra and frequency of known and novel LCA-causative variants in the analysed Japanese cohorts, which were previously unstudied.

Of the 12 (nine LCA-associated and three other IRD-associated) genes found to contain potential pathogenic variants, the most frequently mutated genes were *CRB1* (8.8%, 3/34), *NMNAT1* (8.8%, 3/34), and *RPGRIP1* (8.8%, 3/34) (Fig. 1). All three of the patients (EYE42, EYE159, and S132) found to harbour a missense variant in *NMNAT1* were shown to carry a heterozygous variant c.709C > T [p.(R237C)], and patients EYE159 and S132 were also found to harbour a heterozygous variant c.196C > T [p.(R66W)] (Table 2). While p.(R237C) and p.(R66W) were originally identified as being pathogenic in French, Asian American, and Indian populations^12,13^, Han J *et al*. recently reported two unrelated patients from Korean families with LCA that were compound heterozygous for the two variants^31^. They have since been detected in Japanese families, as well as those of various other ethnicities^6–8,12,13,17,31^. Conversely, nonsense, frameshift, or canonical splice-site variants in *RPGRIP1* were identified in the three of the analysed families (EYE20, EYE55, and EYE149), as was the heterozygous *RPGRIP1* exon-17 deletion (see families EYE16, EYE55, and JIKEI-122) (Table 2). A homozygous *RPGRIP1* exon-17 deletion variant was previously reported in a Japanese family with LCA^25^, but not in any other ethnic populations to date. This, together with the results of the present study, suggests that the *RPGRIP1* exon-17 deletion variant may be a founder mutation in Japanese LCA.

Other genes found to be mutated in the analysed cohort included *GUCY2D* (5.9%, 2/34 families), *CEP290* (2.9%, 1/34 families), *CRX* (2.9%, 1/34 families), *IMPDH1* (2.9%, 1/34 families), *LRAT* (2.9%, 1/34 families), *PRPH2* (2.9%, 1/34 families), *RP2* (2.9%, 1/34 families), *RPGR* (2.9%, 1/34 families), and *BEST1* (2.9%, 1/34 families) (Fig. 1). To the best of our knowledge, this is the first study to report on *CEP290*, *IMPDH1*, *LRAT*, *PRPH2*, *RP2*, *RPGR*, and *BEST1* variants in Japanese families with LCA. *RP2*, *RPGR*, and *BEST1* variants have been previously shown to cause IRDs other than LCA. Only a small number of patients with *RPGR-* or *RP2*-associated LCA have been reported in Chinese cohorts^6,32^; however, there is very limited information regarding patients with *BEST1-*associated LCA^17^. Although the *BEST1* variant p.(D228Y) identified by the present study was shown to be rare and likely pathogenic based on the conducted *in silico* analyses, we did not provide experimental evidences supporting the genotype-phenotype correlations. Thus, it remains possible that the variant(s) in other genes not including our designed panel might be pathogenic in patient EYE187. Further analysis with more large-scale cohort study is necessary to better elucidate the genotype-phenotype correlations for patients with LCA that harbour *BEST1* variants.

The utilised TS approach was not able to detect mutated genes in 15 of the analysed families (Supp. Table 3). It is likely that these 15 families harbour variants in other IRD-, or novel LCA-associated genes that were not targeted by the utilised TS panel; thus, our research group is planning to re-examine these families using a whole exome sequencing (WES) approach. Likewise, the mutation screening data produced by the present study indicated that eight of the 15 unsolved families carry a single rare heterozygous variant in an ar LCA-associated gene (Supp. Table 3), but no second variants were detected via a reanalysis of the generated TS data or conducted CNV analyses, indicating that additional high-throughput sequencing is required. Therefore, we plan to use a whole genome-sequencing approach to screen for these elusive second variants, since they may be located within gene regulatory regions, deep intronic regions, or unknown exons (e.g. alternative splicing regions).

The present study profiled and compared the mutation spectra in Japanese families with LCA to those previously reported in the literature^5–8^. The fact that the study detected a large number of novel variants in the analysed cohort suggests that the genetic basis of Japanese families with LCA is unique. The most frequently mutated genes in European, Chinese, Australian, and Brazilian families with LCA are as follows: European, *CEP290* (15%), *GUCY2D* (11.7%), and *CRB1* (10%)^5^; Chinese, *CRB1* (16.8%), *GUCY2D* (10.7%), and *RPGRIP1* (7.6%)^6^; Brazilian, *CRB1* (10.7%), *CEP290* (10.7%), *RPE65* (10.7%)^7^; Australian, *CEP290* (14.7%), *GUCY2D* (14.7%), and *NMNAT1* (8.8%)^8^. The present study showed that *CRB1* was the most frequently mutated gene among the analysed Japanese families and the families of various other ethnic populations, except previously reported Australian families with LCA. Both the present Japanese, and the previously reported Australian families with LCA exhibited a higher proportion (8.8%) of *NMNAT1* variants than studied Chinese (2.3%) or Brazilian (3.6%) families with LCA. Conversely, the present Japanese cohort exhibited fewer *CEP290* variants (2.9%) than was previously reported to occur in European (15%), Australian (14.7%), and Brazilian (10.7%) families with LCA. Indeed, a large proportion of European, Australian, and Brazilian families with LCA have been shown to carry a founder intronic variant in *CEP290* (c.2991 + 1655G > A), that was not detected by the present study (Supp. Table 4). Together, these data strongly suggest that the mutation spectra in Japanese families with LCA are distinctly different to those characteristic of families with LCA of various other ethnic backgrounds.

The present study employed a TS approach that has been demonstrated to capable of accurately and efficiently identifying variants in patients with IRDs^10,33,34^; however, deep intronic changes and novel genes cannot be identified using this technique. Conversely, the application of WES in genetic diagnosis is aimed for the discovery of novel disease causing genes and WES successfully increases the yield of identification of new causative genes in LCA^20–24^. Although WES will become a standard method for mutation screening in the near future, the utilised TS approach still has a number of advantages. Firstly, this approach can produce a higher coverage rate for targeted regions. Secondly, it can allow more patients to be analysed in a single assay, which leads to less expensive examinations per patient. Thirdly, output data from TS are relatively small and thus make bioinformatics analyses faster and easier. In addition, it reduces the risk of incidental findings. Thus, the TS approach is currently more practically applicable than WES for screening highly heterogeneous diseases such as IRD.

In conclusion, we reported the results of the first NGS-based molecular diagnosis of the largest Japanese LCA cohort so far to our knowledge. We successfully identified potential pathogenic variants in 19 of the 34 analysed families. The mutation spectra and frequency of LCA-associated genes in the Japanese population appears to be distinctly different from those previously reported for other ethnic populations. Finally, the observed detection rate of approximately 56% indicates that the utilised TS approach is a valuable method for diagnosing LCA, and as such, may facilitate the future application of gene-specific treatments for patients with the disease.

## Methods

### Ethics statements

This study was approved by the Institutional Review Board for Human Genetic and Genome Research at the Hamamatsu University School of Medicine (permit no. 14-040), the National Centre for Child Health and Development (permit no. 686), the Jikei University School of Medicine (permit no. 24-232 6997), and the University of Occupational and Environmental Health (permit no. H29-03). All study procedures adhered to the tenets of the Declaration of Helsinki. Written informed consent was obtained either from the participating individuals or from their legal guardians after all the study procedures were explained in detail.

### Patient recruitment

The study participants were recruited from all regions of Japan except the Okinawa islands, and were examined at the Department of Ophthalmology of the National Centre for Child Health and Development Hospital in Tokyo (by NA, SN, and TY), the Department of Ophthalmology at the Jikei University School of Medicine Hospital in Tokyo (by TN, TH, SK, and KM), the Department of Ophthalmology, Hamamatsu University Hospital in Hamamatsu (by YH, MS, AH, KK, and DM), or the University of Occupational and Environmental Health in Fukuoka (by HK). All patients met the following criteria: (1) severe visual impairment (e.g. nyctalopia, nystagmus, very poor or absent ocular pursuit, or oculodigital sign) within the first year after birth; (2) a severely reduced or non-detectable ERG; (3) no systemic abnormality other than neurodevelopmental delay at the time of examination. Clinical diagnoses were based on the results of standard ophthalmic examination techniques including fundus photography, optical coherence tomography, and electroretinography; however, the criteria for classifying LCA varied widely between individuals at the various institutions. Thus, it should be noted that the three listed criteria may not be necessarily sufficient for a standard LCA diagnosis.

### DNA extraction

Genomic DNA was extracted from peripheral-blood leukocytes collected from patients and available family members using the QIAamp DNA Blood Midi Kit (Qiagen, Hilden, Germany), according to the manufacturer’s instructions.

### Comprehensive molecular diagnosis of LCA-causative variants

Comprehensive mutation screening was performed via the series of steps summarized in Supp. Figure 3.

### Target capture and NGS

The integrity of the TS approach used in this study has been previously evaluated using three families that included six patients with LCA^10,11^. These previous results were combined with those presented in the current study, which used a target-capture panel targeted to 74 IRD genes to analyse 33 new patients with LCA (from 31 families). The panel was designed, the library prepared, and the target-capture sequencing performed as previously described^10^.

### Bioinformatics analyses

The sequence reads were mapped to the human reference genome sequence (GRCh37/hg19) using Burrows-Wheeler Aligner software (v 0.7.15) after trimming the adapter sequence by cutadapt software (v 1.11) and mapped reads around insertion-deletion polymorphisms (INDELs) were realigned by Genome Analysis Toolkit (GATK; v 3.6)^35^. Base quality scores were recalibrated by GATK. Variant calls were processed by the GATK HaplotypeCaller, and called single nucleotide variants and INDELs were annotated by ANNOVAR software (v 2016Feb01)^36^. We focused on nonsynonymous variants and splice site variants which are within 5 bp of the exon-intron boundaries (±5 bp), and excluded synonymous and non-coding exonic variants for the analysis. Common genetic variants (allele frequency, >0.005 for recessive variants or >0.001 for dominant variants) in any of the ethnic subgroups found in the following single nucleotide polymorphism (SNP) databases and synonymous variants were treated as possible non-pathogenic sequence alterations in this study: 1000 Genomes database (http://www.1000genomes.org/), Exome Aggregation Consortium database (http://exac.broadinstitute.org/), Human Genetic Variation Database (HGVD; http://www.genome.med.kyoto-u.ac.jp/SnpDB/) and Tohoku Medical Megabank Organization database (ToMMo; https://ijgvd.megabank.tohoku.ac.jp/). HGVD and ToMMo were used as a reference for Japanese controls. The Human Gene Mutation Database (HGMD; https://portal.biobase-international.com/cgi-bin/portal/login.cgi) was used to identify previously reported variants. A bioinformatics analysis of the analysed region was conducted to exclude false positive variants; however, this may have also filtered out true pathogenic variants. Therefore, for those families in which the utilized TS approach only identified a heterozygous rare variant in ar LCA-associated genes, we expanded the analysed region, and re-examined the our TS data to screen for variants located in intronic regions within 25 bp of the exon-intron boundaries (±25 bp)^10^, and non-coding exonic regions^30^.

### Prioritization and assessment of identified variants

Identified variants were filtered by applying the following prioritization criteria:

1. Variants present in the HGMD were considered to be known pathogenic mutations.
2. Nonsense, frameshift, or canonical splice-site variants were considered to be likely pathogenic.
3. Missense variants were considered to be potentially pathogenic when at least two *in silico* computational algorithms, (as applied using either SIFT [http://sift.jcvi.org/www/SIFT_seq_submit2.html], PolyPhen2 [http://genetics.bwh.harvard.edu/pph2/], Mutation Taster [http://www.mutationtaster.org/], or CADD [http://cadd.gs.washington.edu/] software), produced a positive score. Splice-site variants were similarly analysed using Neural Network Splice Site Prediction software (http://www.fruitfly.org/seq_tools/splice.html).
4. We then screened the remaining variants that matched the patients’ phenotypes and the reported inheritance patterns of the respective genes.

Pathogenicity was considered according to the ACMG guidelines^37^.

### Additional mutation screening

Families for whom no potentially pathogenic variant(s) were identified via the utilised TS method underwent additional mutation screening, as below (Supp. Table 4):

1. The known *CEP290* intronic variant c.2991 + 1655A > G^38^ was not included in the design of the TS capture panel, so genomic *CEP290* fragments encompassing c.2991 + 1655A > G had to be amplified and analysed via Sanger sequencing.
2. Large deletion and insertion variants were not detectable via the applied TS approach; thus, a long-range PCR assay was used to screen the known *RPGRIP1* exon 17 deletion^11,25^.
3. The LCA-associated genes *CCT2*, *CLUAP1*, *DTHD1*, *GDF6*, and *IFT140*^20–24^ were identified after the capture panel used in the present study was designed (as reported in RetNet at the time of system design; https://sph.uth.edu/retnet/; accessed 23th January 2014). Thus, all coding exons in *GDF6* and *IFT140* were screened via Sanger sequencing. Given the rarity of reported LCA-associated variants in *CCT2*, *CLUAP1*, and *DTHD1*, only those exons within these genes that contained previously reported variants were analysed.
4. The *RPGR* exon ORF15 is a mutational hotspot for X-linked RP; however, it contains repetitive sequences that cannot be efficiently captured or enriched via conventional targeted capture NGS^39^. Thus, genomic *RPGR* fragments encompassing the ORF15 region were amplified and analysed via Sanger sequencing for the unsolved male patients with LCA (i.e. EYE16, EYE103, EYE133, EYE178, EYE182, and JU0955).

### CNV analyses

Screening for CNV in identified ar LCA-associated genes was performed for families in which the utilised TS or additional mutation screening approaches revealed only a single heterozygous rare variant (Supp. Table 3). SALASA MLPA probemix reagents (P221 for *CRX*, and P222 for *RPGRIP1*, *GUCY2D*, and *CEP290;* MRC Holland, Amsterdam, Netherland) were used to conduct an MLPA, as previously described^40^. A qPCR analysis was performed to confirm the family JIKEI-145 segregation analysis findings, using appropriate primers (available upon request), SYBR Premix EX Taq II (Takara, Japan) and a Thermal Cycler Dice TP800 (Takara, Japan), according to the manufacturer’s instructions. Relative comparative threshold cycle (Ct) values were calculated on the basis of the second derivative maximum method using dedicated software (Thermal Cycler Dice Real Time System software, v 5.11B for TP800, Takara, Japan). The relative copy number (RCN) was determined on the basis of the comparative ddCT method using the unaffected father DNA as a control (where an RCN score of 1.0 represented no copy number change, a score of 0.6–1.4 was considered abnormal, and a score <0.6 and >1.4 represented deletions and duplications, respectively). All reactions were performed in duplicate, and all experiments were repeated in triplicate.

### Sanger sequencing and segregation analyses

Potential pathogenic variants detected using the TS approach were validated by performing Sanger sequencing as per the standard protocol^41^. All utilised primers are available upon request. Sanger sequencing segregation analyses were performed on DNA from family members to investigate the co-segregation of potentially pathogenic variants.

### Supplementary data

Supplementary data include three figures and four tables^10,13–15,25,26,42–47^.

## Electronic supplementary material

Supplementary information
