# Supplementary material for: Molecular Diagnosis of 34 Japanese Families with Leber Congenital Amaurosis Using Targeted Next Generation Sequencing
Source: Sci Rep. 2018 May 29;8:8279. doi: 10.1038/s41598-018-26524-z (PMC5974356; doi:10.1038/s41598-018-26524-z)
Supplement: Supplementary file 1 — Supplementary information [file 41598_2018_26524_MOESM1_ESM.docx]

**Molecular Diagnosis of 34 Japanese Families with Leber Congenital** **Amaurosis Using Targeted Next Generation Sequencing**

Katsuhiro Hosono^1^, Sachiko Nishina^2^, Tadashi Yokoi^2^, Satoshi Katagiri^2,3^, Hirotomo Saitsu^4^, Kentaro Kurata^1^, Daisuke Miyamichi^1^, Akiko Hikoya^1^, Kei Mizobuchi^3^, Tadashi Nakano^3^, Shinsei Minoshima^5^, Maki Fukami^6^, Hiroyuki Kondo^7^, Miho Sato^1^, Takaaki Hayashi^3^, Noriyuki Azuma^2^, Yoshihiro Hotta^1*^

^1^Department of Ophthalmology, Hamamatsu University School of Medicine, Shizuoka, Japan

^2^Department of Ophthalmology and Laboratory for Visual Science, National Centre for Child Health and Development, Tokyo, Japan

^3^Department of Ophthalmology, The Jikei University School of Medicine, Tokyo, Japan

^4^Department of Biochemistry, Hamamatsu University School of Medicine, Shizuoka, Japan

^5^Department of Photomedical Genomics, Institute for Medical Photonics Research, Preeminent Medical Photonics Education & Research Centre, Hamamatsu University School of Medicine, Shizuoka, Japan

^6^Department of Molecular Endocrinology, National Centre for Child Health and Development, Tokyo, Japan

^7^Department of Ophthalmology, University of Occupational and Environmental Health, Fukuoka, Japan

***Corresponding author**: Yoshihiro Hotta

**Supplementary Table 1**. Target coverage for each gene in the present study.

| **Gene** | **Coverage** |  | **Gene** | **Coverage** |  | **Gene** | **Coverage** |
| --- | --- | --- | --- | --- | --- | --- | --- |
| *ABCA4* | 99.97% |  | *IMPG2* | 99.72% |  | *RBP3* | 100% |
| *AIPL1* | 95.75% |  | *IQCB1* | 99.87% |  | *RD3* | 100% |
| *ARL2BP* | 99.56% |  | *KCNJ13* | 100% |  | *RDH12* | 100% |
| *BEST1* | 100% |  | *KIAA1549* | 99.67% |  | *RGR* | 93.58% |
| *C2orf71* | 100% |  | *KLHL7* | 100% |  | *RHO* | 100% |
| *C8orf37* | 100% |  | *LCA5* | 99.91% |  | *RLBP1* | 100% |
| *CA4* | 97.17% |  | *LRAT* | 98.64% |  | *ROM1* | 100% |
| *CABP4* | 98.52% |  | *MAK* | 99.61% |  | *RP1* | 99.92% |
| *CEP290* | 99.53% |  | *MERTK* | 100% |  | *RP2* | 98.38% |
| *CERKL* | 98.75% |  | *MVK* | 100% |  | *RP9* | 92.88% |
| *CLRN1* | 100% |  | *NEK2* | 99.70% |  | *RPE65* | 98.34% |
| *CNGA1* | 99.91% |  | *NMNAT1* | 93.99% |  | *RPGR* | 89.70% |
| *CNGB1* | 100% |  | *NR2E3* | 100% |  | *RPGRIP1* | 100% |
| *CRB1* | 99.71% |  | *NRL* | 99.24% |  | *SAG* | 97.70% |
| *CRX* | 96.97% |  | *OFD1* | 100% |  | *SEMA4A* | 100% |
| *DHDDS* | 99.86% |  | *OTX2* | 100% |  | *SNRNP200* | 97.71% |
| *DTHD1* | 99.57% |  | *PDE6A* | 98.76% |  | *SPATA7* | 98.93% |
| *EMC1* | 99.99% |  | *PDE6B* | 99.60% |  | *TOPORS* | 100% |
| *EYS* | 95.78% |  | *PDE6G* | 100% |  | *TTC8* | 100% |
| *FAM161A* | 99.63% |  | *PRCD* | 100% |  | *TULP1* | 100% |
| *FSCN2* | 100% |  | *PROM1* | 100% |  | *USH2A* | 100% |
| *ADGRA3* | 99.60% |  | *PRPF3* | 98.45% |  | *ZNF513* | 100% |
| *GUCA1B* | 96.28% |  | *PRPF31* | 100% |  |  |  |
| *GUCY2D* | 98.44% |  | *PRPF6* | 100% |  |  |  |
| *IDH3B* | 100% |  | *PRPF8* | 99.79% |  |  |  |
| *IMPDH1* | 100% |  | *PRPH2* | 100% |  |  |  |

Using the Agilent SureDesign (https://earray.chem.agilent.com/suredesign/index.htm), a custom target enrichment library was designed to capture the 74 genes known to be associated with RP or LCA, as reported in the RetNet (https://sph.uth.edu/retnet/) accessed on 23th January 2014. The target coverage for each gene is shown.

**Supplementary Table 2.** Results of the *in silico* analyses of the identified novel missense variants.

|  |  |  | Predicted effect of variant* | | | |
| --- | --- | --- | --- | --- | --- | --- |
| Patient | Affected gene | Variant | SIFT | PolyPhen2 | Mutation Taster | CADD |
| EYE50 | *RPGR* | c.977A>C;p.(K326T) | Damaging (0.002) | Probably Damaging (1.000) | Disease causing (1.000) | 24.5 |
| EYE121 | *CRB1* | c.2T>C;p.? | Damaging (0.000) | Possibly Damaging (0.745) | Disease causing (1.000) | 19.4 |
| EYE121 | *CRB1* | c.3068T>G;p.(L1023R) | Damaging (0.001) | Probably Damaging (1.000) | Disease causing (1.000) | 24.1 |
| EYE125 | *IMPDH1* | c.590A>C;p.(Q197P) | Damaging (0.035) | Benign (0.000) | Disease causing (1.000) | 22.1 |
| EYE139 | *GUCY2D* | c.2765A>G;p.(Y922C) | Damaging (0.000) | Probably Damaging (1.000) | Disease causing (1.000) | 27.0 |
| EYE156 | *PRPH2* | c.748T>G;p.(C250G) | Damaging (0.000) | Probably Damaging(1.000) | Disease causing (1.000) | 26.8 |
| EYE187 | *BEST1* | c.682G>T;p.(D228Y) | Damaging (0.000) | Probably Damaging(1.000) | Disease causing (1.000) | 33.0 |

*Identified missense variants were considered to be potentially pathogenic if they were awarded a positive score via assessment with at least two separate *in silico* computational algorithms, using either SIFT (http://sift.jcvi.org/www/SIFT_seq_submit2.html), PolyPhen2 (http://genetics.bwh.harvard.edu/pph2/), Mutation Taster (http://www.mutationtaster.org/), and/or CADD (http://cadd.gs.washington.edu/) software.

**Supplementary Table 3**. Summary of the potential pathogenic variants and rare variants in 34 Japanese families with Leber Congenital Amaurosis (LCA).

|  |  |  |  |  |  |  |  |  | Databases^a^ | | | |  |  |
| --- | --- | --- | --- | --- | --- | --- | --- | --- | --- | --- | --- | --- | --- | --- |
| Family ID | Patient ID | Gen -der | Analysis methods | Gene | Nucleotide change | Zygosity | Segregation analysis | SNP ID | HGVD | ToMMo | 1000 Genomes | ExAC | **ACMG classifi-cation** | Reference |
| EYE16 | EYE16^c^ | M | TS, AS2, ML | *TULP1* | c.1145T>C;p.(F382S) | Het |  |  | 0.0009 | 0.001 | 0 | 1.95 × 10^-5^ | L.P | 26 |
|  |  |  |  | *RPGRIP1* | **Exon 17 deletion (c.2710+374_2895+74del)** | Het |  |  | 0 | 0 | 0 | 0 | P | 25 |
| EYE17 | EYE17 | F | TS, AS1 | *MAK*^d^ | c.565A>G;p.(I189V) | Het |  | rs56215624 | 0.0014 | 0.0022 | 0 | 3.30 × 10^-5^ | U.S | Novel |
| EYE20 | EYE20 | F | TS | *RPGRIP1* | **c.3565_3571delCGAAGGC;p.(R1189Gfs*7)** | Hom | Biparentally |  | 0 | 0 | 0 | 1.66 × 10^-5^ | P | 42 |
|  | EYE64 | M | TS | *RPGRIP1* | **c.3565_3571delCGAAGGC;p.(R1189Gfs*7)** | Hom | Biparentally |  | 0 | 0 | 0 | 1.66 × 10^-5^ | P | 42 |
|  | EYE65 | M | TS | *RPGRIP1* | **c.3565_3571delCGAAGGC;p.(R1189Gfs*7)** | Hom | Biparentally |  | 0 | 0 | 0 | 1.66 × 10^-5^ | P | 42 |
| EYE42 | EYE42 | M | TS | *NMNAT1* | **c.1A>G;p.?** | Het | Maternal |  | 0 | 0 | 0 | 8.26 × 10^-6^ | P | 13 |
|  |  |  |  | *NMNAT1* | **c.709C>T;p.(R237C)** | Het | Paternal | rs375110174 | 0.0009 | 0.0002 | 0.0000 | 7.42 × 10^-5^ | P | 12,13 |
|  |  |  |  | *RBP3*^d^ | c.1807G>A;p.G603S | Het |  | rs202017297 | 0 | 0.0005 | 0.0002 | 3.47 × 10^-5^ | U.S | Novel |
| EYE47 | EYE47 | F | TS, AS1 | *RD3* | c.89T>A;p.(M30K) | Het |  |  | 0.0014 | 0.0007 | 0 | 3.31 × 10^-5^ | U.S | Novel |
|  |  |  |  | *CEP290* | c.5884A>T;p.(R1962W) | Het |  |  | 0 | 0 | 0 | 0 | U.S | Novel |
|  |  |  |  | *AIPL1* | c.284G>A;p.(G95E) | Het |  | rs563684228 | 0.0005 | 0 | 0.0002 | 0 | U.S | Novel |
| EYE50 | EYE50 | M | TS | *RPGR* | **c.977A>C;p.(K326T)** | Hemi | Maternal |  | 0 | 0 | 0 | 0 | L.P | Novel |
| EYE55 | EYE55 | M | TS, AS2 | *RPGRIP1* | **c.1467+1G>T** | Het | Paternal |  | 0 | 0 | 0 | 0 | P | 11 |
|  |  |  |  | *RPGRIP1* | **Exon 17 deletion (c.2710+374_2895+74del)** | Het | Maternal |  | 0 | 0 | 0 | 0 | P | 11,25 |
| EYE63 | EYE63^c^ | F | TS, AS1, ML | *RPGRIP1* | c.1802C>G;p.(S601W) | Het |  | rs3748360 | 0.0023 | 0 | 0.0002 | 4.14 × 10^-5^ | U.S | Novel |
|  |  |  |  | *GUCY2D* | c.2704G>C;p.(V902L) | Het |  |  | 0 | 0 | 0 | 0 | L.P | Novel |
| EYE68 | EYE68 | M | TS | *CRB1* | **c.668dupT;p.(L223Ffs*4)** | Het | Maternal |  | 0 | 0 | 0 | 0 | P | Novel |
|  |  |  |  | *CRB1* | **c.733dupG;p.(A245Gfs*16)** | Het | Maternal |  | 0 | 0 | 0 | 0 | P | Novel |
|  |  |  |  | *CRB1* | **c.1567dupC;p.(L523Pfs*28)** | Het | Paternal |  | 0 | 0 | 0 | 0 | P | Novel |
| EYE69 | EYE69 | M | TS | *CEP290* | **c.2390delA;p.(K797Sfs*2)** | Het | Maternal | rs781670422 | 0 | 0 | 0 | 5.10 × 10^-5^ | P | 43 |
|  |  |  |  | *CEP290* | **c.6889A>T;p.K2297*** | Het | Paternal |  | 0 | 0 | 0 | 0 | P | Novel |
|  |  |  |  | *SPATA7* | c.890A>T;p.(D297V) | Het |  | rs769211713 | 0.0044 | 0.0034 | 0 | 8.73 × 10^-6^ | U.S | Novel |
|  |  |  |  | *CERKL*^d^ | c.1438A>T;p.(N480Y) | Het |  |  | 0.0005 | 0 | 0 | 0 | U.S | Novel |
| EYE70 | EYE70 | F | TS | *CRX* | **c.124G>A;p.(E42K)** | Het | *de novo* | rs863224863 | 0 | 0 | 0 | 0 | P | 15 |
|  |  |  |  | *KIAA1549*^d^ | c.5198A>G;p.(Q1733R) | Het |  |  | 0.0019 | 0 | 0 | 0 | U.S | Novel |
| EYE103 | EYE103 | M | TS, AS2 | *EYS*^d^ | c.525_527del;p.(E176del) | Het |  |  | 0 | 0 | 0 | 0.0001 | L.P | Novel |
|  |  |  |  | *CNGB1*^d^ | c.1696G>A;p.(D566N) | Het |  |  | 0 | 0.0002 | 0 | 2.49 × 10^-5^ | U.S | Novel |
| EYE114 | EYE114 | M | TS | *RP2* | **c.769-2A>G** | Hemi | Maternal |  | 0 | 0 | 0 | 0 | P | Novel |
| EYE115 | EYE115 | F | TS | *CRB1* | **c.1334_1740del;p.(C445Yrfs*8)** | Het | Maternal |  | 0 | 0 | 0 | 0 | P | Novel |
|  |  |  |  | *CRB1* | **c.1576C>T;p.(R526*)** | Het | Paternal | rs114342808 | 0 | 0.0002 | 0 | 4.94 × 10^-5^ | P | 42 |
| EYE120 | EYE120 | F | TS, AS1 |  | Rare variant was not found |  |  |  |  |  |  |  |  |  |
| EYE121 | EYE121 | F | TS | *CRB1* | **c.2T>C;p.?** | Het |  |  | 0 | 0 | 0 | 0 | P | Novel |
|  |  |  |  | *CRB1* | **c.3068T>G;p.(L1023R)** | Het | Maternal |  | 0 | 0.0002 | 0 | 0 | L.P | Novel |
|  |  |  |  | *KIAA1549*^d^ | c.3164C>T;p.(P1055L) | Het |  | rs368584498 | 0 | 0 | 0 | 2.84 × 10^-5^ | U.S | Novel |
| EYE125 | EYE125 | M | TS | *IMPDH1* | **c.590A>C;p.(Q197P)** | Het | *de novo* |  | 0 | 0 | 0 | 0 | L.P | Novel |
| EYE133 | EYE133 | M | TS, AS2 |  | Rare variant was not found |  |  |  |  |  |  |  |  |  |
| EYE139 | EYE139 | M | TS | *GUCY2D* | **c.2765A>G;p.(Y922C)** | Het | Maternal |  | 0 | 0.0003 | 0 | 0 | L.P | Novel |
|  |  |  |  | *GUCY2D* | **c.2983C>T;p.(R995W)** | Het | Paternal | rs61750187 | 0 | 0 | 0 | 0 | P | 14 |
|  |  |  |  | *RPGRIP1* | c.3835_3837delGAG;p.(E1279del) | Het |  | rs281865293 | 0 | 0 | 0 | 0.0002 | U.S | 44 |
|  |  |  |  | *EMC1*^d^ | c.1169C>T;p.(T390M) | Het |  | rs144716849 | 0.0005 | 0 | 0.0046 | 0.0013 | U.S | Novel |
| EYE149 | EYE149 | M | TS | *RPGRIP1* | **c.799C>T;p.(R267*)** | Het | Paternal | rs554396590 | 0 | 0 | 0.0002 | 8.36 × 10^-6^ | P | 15 |
|  |  |  |  | *RPGRIP1* | **c.1687C>T;p.(R563*)** | Het | Maternal |  | 0 | 0 | 0 | 0 | P | Novel |
|  |  |  |  | *USH2A*^d^ | c.5608C>T;p.(R1870W) | Het |  | rs144768593 | 0.0023 | 0.0041 | 0.0004 | 0.0004 | U.S | 46 |
|  |  |  |  | *C2orf71*^d^ | c.85C>T;p.(R29W) | Het |  | rs201706430 | 0.0027 | 0.0037 | 0.0002 | 0.0003 | U.S | 45 |
| EYE152 | EYE152^c^ | F | TS, AS2, ML | *RPGRIP1* | c.2554C>T;p.(R852*) | Het |  |  | 0 | 0 | 0 | 0 | P | Novel |
|  |  |  |  | *EMC1*^d^ | c.1187A>C;p.(E396A) | Het |  |  | 0 | 0 | 0 | 0 | U.S | Novel |
| EYE156 | EYE156 | F | TS | *PRPH2* | **c.730_736delinsCAGCTCCTCCAGACGGGTGCACCAGAC;p.(N244Qfs*19)** | Het | Maternal |  | 0 | 0 | 0 | 0 | P | Novel |
|  |  |  |  | *PRPH2* | **c.748T>G;p.(C250G)** | Het | Paternal |  | 0 | 0 | 0 | 0 | L.P | Novel |
|  |  |  |  | *MAK*^d^ | c.1205A>C;p.(K402T) | Het |  | rs200533678 | 0 | 0.0005 | 0 | 4.94 × 10^-5^ | U.S | Novel |
| EYE159 | EYE159 | M | TS | *NMNAT1* | **c.196C>T;p.(R66W)** | Het | Maternal |  | 0 | 0.0002 | 0 | 0.0001 | P | 12 |
|  |  |  |  | *NMNAT1* | **c.709C>T;p.(R237C)** | Het | Paternal | rs375110174 | 0.0009 | 0.0002 | 0 | 7.42 × 10^-5^ | P | 12,13 |
|  |  |  |  | *ABCA4*^d^ | c.5714+4C>T | Het |  | rs376586802 | 0 | 0 | 0 | 4.97 × 10^-5^ |  | 47 |
| EYE170 | EYE170^c^ | F | TS, AS2, ML | *RPGRIP1* | **c.3565_3571delCGAAGGC;p.(R1189Gfs*7)** | Het |  |  | 0 | 0 | 0 | 1.66 × 10^-5^ | P | 42 |
| EYE178^b^ | EYE178^c^ | M | TS, AS2, ML | *CRX* | c.225G>C;p.(K75N) | Het | Maternal |  | 0 | 0 | 0 | 0 | U.S | Novel |
| EYE181 | EYE181 | F | TS, AS1 |  | Rare variant was not found |  |  |  |  |  |  |  |  |  |
| EYE182 | EYE182 | M | TS, AS2 |  | Rare variant was not found |  |  |  |  |  |  |  |  |  |
| EYE187 | EYE187 | M | TS | *BEST1* | **c.682G>T;p.(D228Y)** | Het | Paternal  (mosaic)^e^ |  | 0 | 0 | 0 | 0 | L.P | Novel |
| JIKEI-086 | JU0754 | F | TS, AS1 |  | Rare variant was not found |  |  |  |  |  |  |  |  | Novel |
| JIKEI-122 | JU0954 | F | TS, AS1, ML | *RPGRIP1* | **Exon 17 deletion (c.2710+374_2895+74del)** | Het |  |  | 0 | 0 | 0 | 0 | P | 25 |
|  |  |  |  | *ABCA4*^d^ | c.4715C>T;p.(T1572M) | Het |  | rs185093512 | 0.0009 | 0.0024 | 0 | 0.0001 | U.S | Novel |
|  | JU0955 | M | TS, AS2, ML | *RPGRIP1* | **Exon 17 deletion (c.2710+374_2895+74del)** | Het |  |  | 0 | 0 | 0 | 0 | P | 25 |
|  |  |  |  | *ABCA4*^d^ | c.4715C>T;p.(T1572M) | Het |  | rs185093512 | 0.0009 | 0.0024 | 0 | 0.0001 | U.S | Novel |
| JUKEI-145 | JU1039 | F | TS, QP | *LRAT* | **c.163C>T;p.(R55W)** | Het | Paternal | rs527236079 | 0.0018 | 0.0025 | 0 | 0 | P | 33 |
|  |  |  |  | *LRAT* | **Exon 1–3 deletion** | Het | Maternal |  | 0 | 0 | 0 | 0 | P | Novel |
|  | JU1040 | F | TS, QP | *LRAT* | **c.163C>T;p.(R55W)** | Het | Paternal | rs527236079 | 0.0018 | 0.0025 | 0 | 0 | P | 33 |
|  |  |  |  | *LRAT* | **Exon 1–3 deletion** | Het | Maternal |  | 0 | 0 | 0 | 0 | P | Novel |
| S132 | S132 | F | TS | *NMNAT1* | **c.196C>T;p.R66W** | Het | Paternal |  | 0 | 0.0002 | 0 | 0.0001 | P | 12 |
|  |  |  |  | *NMNAT1* | **c.709C>T;p.(R237C)** | Het | Maternal | rs375110174 | 0.0009 | 0.0002 | 0 | 7.42 × 10^-5^ | P | 12,13 |
|  |  |  |  | *RPGRIP1* | c.1802C>G;p.(S601W) | Het |  | rs3748360 | 0.0023 | 0 | 0.0002 | 4.14 × 10^-5^ | U.S | Novel |
|  |  |  |  | *SPATA7* | c.890A>T;p.(D297V) | Het |  | rs769211713 | 0.0044 | 0.0034 | 0 | 8.73 × 10^-6^ | U.S | Novel |
|  |  |  |  | *PDE6B*^d^ | c.1679G>A;p.(R560H) | Het |  |  | 0.0044 | 0.0034 | 0 | 6.60 × 10^-5^ | U.S | Novel |
| LCA1H | LCA1H (Twin 1) | M | TS | *GUCY2D* | **c.2113+2_2113+3insT** | Het | Paternal |  | 0 | 0 | 0 | 0 | P | 10 |
|  |  |  |  | *GUCY2D* | **c.2714T>C;p.(L905P)** | Het | Maternal |  | 0 | 0 | 0 | 0 | P | 10 |
|  | LCA1H (Twin 2) | M | TS | *GUCY2D* | **c.2113+2_2113+3insT** | Het | Paternal |  | 0 | 0 | 0 | 0 | P | 10 |
|  |  |  |  | *GUCY2D* | **c.2714T>C;p.(L905P)** | Het | Maternal |  | 0 | 0 | 0 | 0 | P | 10 |
| LCA2H | LCA2H^c^ | F | TS, AS1, ML | *CEP290* | c.1345A>G;p.(K449E) | Het |  | rs201527662 | 0.0009 | 0.0020 | 0.0008 | 0.0002 | U.S | Novel |

Bold symbols indicate potential pathogenic variants.

^a^1000 Genomes database (1000 Genomes; http://www.1000genomes.org/), Exome Aggregation Consortium database (ExAC; http://exac.broadinstitute.org/), Human Genetic Variation Database (HGVD; http://www.genome.med.kyoto-u.ac.jp/SnpDB/) and Tohoku Medical Megabank Organization database (ToMMo; https://ijgvd.megabank.tohoku.ac.jp/).

^b^EYE178 harboured a novel *CRX* variant p.(K75N). *CRX* mutations have been shown to cause autosomal dominant (ad) or recessive (ar) LCA. The unaffected parents in EYE178 were not consanguineous. The conducted segregation analysis indicated that the p.(K75N) variant was maternally inherited and not *de novo*; thus, it is more likely subject to an ar than an ad mode of inheritance.

^c^A multiplex ligation-dependent probe amplification analysis (ML) was performed for eight patients for whom only a single heterozygous rare ar LCA-associated variant was identified, but did not detect a copy number variant in any patient.

^d^Identified rare variants in other IRD-associated genes were not confirmed by Sanger sequencing.

^e^The results of the EYE187 family segregation analysis suggested that the father may harbour the identified variant in a mosaic state, (See Supp. Fig 2).

M, Male; F, Female; TS, Targeted next-generation sequencing; AS1, Additional mutation screening methods 1–3 (See Methods); AS2, Additional mutation screening methods 1–4 (See Methods); QP, Quantitative real-time polymerase chain reaction; Hom, Homozygous; Het, Heterozygous; Hemi, Hemizygous; P, Pathogenic; L.P, Likely pathogenic; U.S, uncertain significance.

**Supplementary Table 4.** Results of the additional mutation screening of unsolved families with LCA.

| Analysed regions or mutations | Reference | Accession number | Patients (n) | Results |
| --- | --- | --- | --- | --- |
| c.2991+1655A>G in *CEP290* | 38 | NM_025114.3 | 16 patients* | Not found |
| Exon 17 deletion in *RPGRIP1* | 25 | NM_020366.3 | 16 patients* | EYE16, JU0954, and JU0955 had the heterozygous variant |
| c.1198A>C and c.1547G>A in *CCT2* | 23 | NM_006431.2 | 16 patients* | Not found |
| c.817C>T in *CLUAP1* | 24 | NM_015041.2 | 16 patients* | Not found |
| c.2T>C in *DTHD1* | 20 | NM_001136536.4 | 16 patients* | Not found |
| Exon 1–2 in *GDF6* | 21 | NM_001001557.2 | 16 patients* | Rare variant was not identified |
| Exon 3–31 in *IFT140* | 22 | NM_014714.3 | 16 patients* | Rare variant was not identified |
| ORF15 in *RPGR* | 39 | NM_001034853.1 | 6 patients** | Rare variant was not identified |

*Mutation screening was performed for seven genes for patients EYE16, EYE17, EYE47, EYE63, EYE103, EYE120, EYE133, EYE152, EYE170, EYE178, EYE181, EYE182, JU10754, JU0954, JU0955 and LCA2H.

**Mutation screening for *RPGR* exon ORF15 was conducted for patients EYE16, EYE103, EYE133, EYE178, EYE182, and JU0955.





**Supplementary Figure 1. Family JIKEI-145 qPCR analysis results.**

The height of the displayed columns represents the relative allelic dosage of the specified genomic segments, such that a value of one corresponds with the presence of two alleles. The X-axis shows the location of the specified region within the *LRAT* sequence. A relative copy number score of 1.0 represents no copy number change, a score of 0.6–1.4 is considered abnormal, and scores < 0.6 and ˃ 1.4 represent deletions and duplications, respectively.


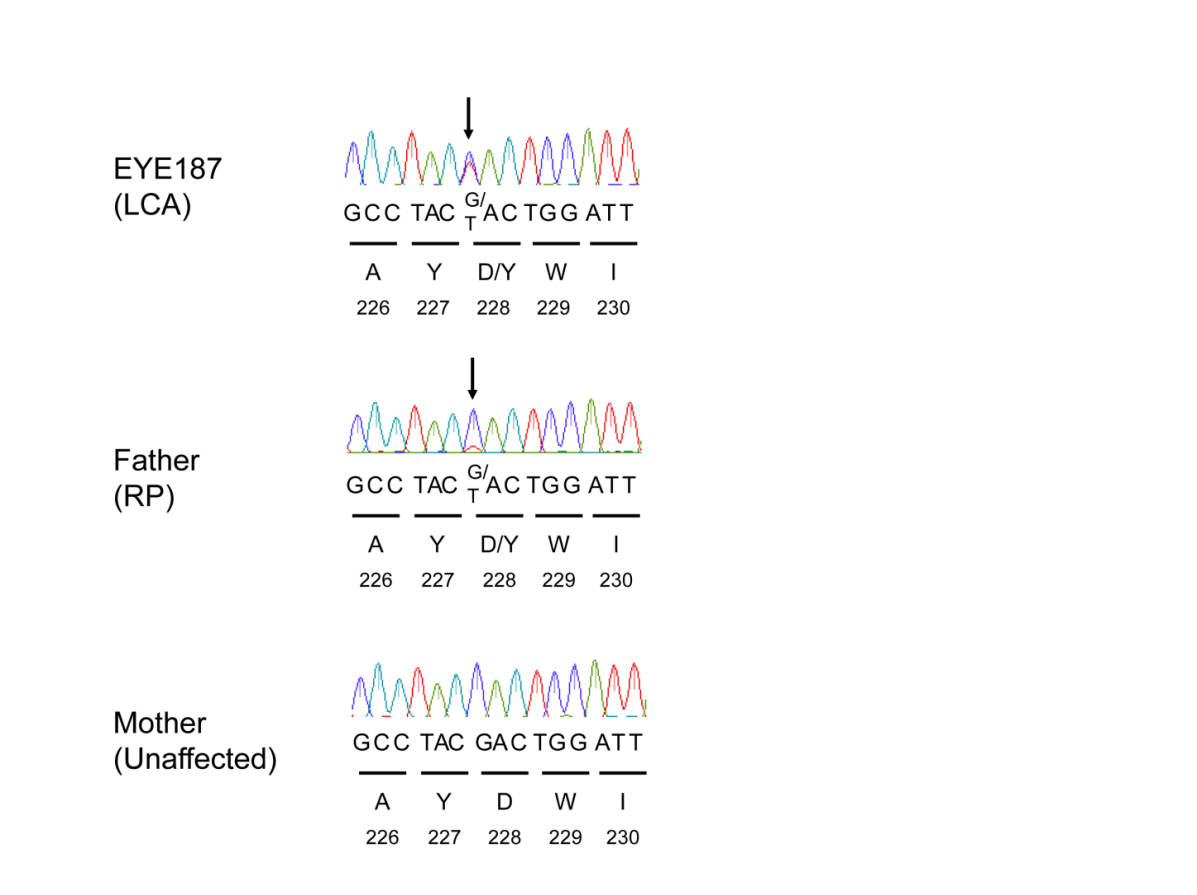


**Supplementary Figure 2. Family EYE187 electropherograms.**

Results of the family EYE187 segregation analysis suggested that the father may harbour the variant in a mosaic state, as shown by the displayed sequencing electropherogram. The mother was normally sighted, while the father had received a diagnosis of retinitis pigmentosa (RP). Deduced amino acids are indicated under the sequence trace. The location of the c.682G>T [p.(D228Y)] variant is indicated by the arrow.





**Supplementary Figure 3. Comprehensive mutation screening flowchart.**

Dotted arrows indicate rare variants that were not detectable in the present cohort via the conducted TS data reanalysis, additional mutation screening, or copy number variation (CNV) analyses. The three specified patient criteria are described in the Methods section.
